# Immune microenvironment of cholangiocarcinoma: Biological concepts and treatment strategies

**DOI:** 10.3389/fimmu.2023.1037945

**Published:** 2023-04-17

**Authors:** Xianzhe Yu, Lingling Zhu, Ting Wang, Jiang Chen

**Affiliations:** ^1^ Lung Cancer Center, West China Hospital of Sichuan University, Chengdu, Sichuan, China; ^2^ Department of Gastrointestinal Surgery, Chengdu Second People’s Hospital, Chengdu, Sichuan, China; ^3^ Department of General Surgery, Sir Run Shaw Hospital, Zhejiang University, Hangzhou, Zhejiang, China

**Keywords:** cholangiocarcinoma, tumor microenvironment, immunotherapy, immune checkpoint blockade, immunosuppressive

## Abstract

Cholangiocarcinoma is characterized by a poor prognosis with limited treatment and management options. Chemotherapy using gemcitabine with cisplatin is the only available first-line therapy for patients with advanced cholangiocarcinoma, although it offers only palliation and yields a median survival of < 1 year. Recently there has been a resurgence of immunotherapy studies focusing on the ability of immunotherapy to inhibit cancer growth by impacting the tumor microenvironment. Based on the TOPAZ-1 trial, the US Food and Drug Administration has approved the combination of durvalumab and gemcitabine with cisplatin as the first-line treatment of cholangiocarcinoma. However, immunotherapy, like immune checkpoint blockade, is less effective in cholangiocarcinoma than in other types of cancer. Although several factors such as the exuberant desmoplastic reaction are responsible for cholangiocarcinoma treatment resistance, existing literature on cholangiocarcinoma cites the inflammatory and immunosuppressive environment as the most common factor. However, mechanisms activating the immunosuppressive tumor microenvironment contributing to cholangiocarcinoma drug resistance are complicated. Therefore, gaining insight into the interplay between immune cells and cholangiocarcinoma cells, as well as the natural development and evolution of the immune tumor microenvironment, would provide targets for therapeutic intervention and improve therapeutic efficacy by developing multimodal and multiagent immunotherapeutic approaches of cholangiocarcinoma to overcome the immunosuppressive tumor microenvironment. In this review, we discuss the role of the inflammatory microenvironment-cholangiocarcinoma crosstalk and reinforce the importance of inflammatory cells in the tumor microenvironment, thereby highlighting the explanatory and therapeutic shortcomings of immunotherapy monotherapy and proposing potentially promising combinational immunotherapeutic strategies.

## 1 Introduction

Cholangiocarcinoma (CCA) is the second most common malignant liver cancer with a poor and short-term incurable prognosis (1). According to their anatomical location, CCAs are categorized as intrahepatic (iCCA), perihilar (pCCA), and distal (dCCA), also referred to as extrahepatic CCA (eCCA), all of which pose additional challenges for clinicians as they feature distinct microenvironments and resistance to treatment (1), thus necessitating different management approaches. Due to the ‘silent’ clinical characteristic of iCCA, diagnosis at an early stage remains a challenge, and late-stage iCCA renders curative surgical resection an unviable treatment option. Chemotherapy, using gemcitabine and cisplatin (Gem+Cis), is the only approved treatment for advanced CCA (2). However, most patients have poor outcomes, and those who do respond, develop resistance to chemotherapy over the course of treatment. Furthermore, there is no second-line standard treatment available for effective systemic therapy for advanced CCA. Advances in genetic profiling and classification have helped develop a multitude of molecularly targeted agents, many of which have entered clinical trial testing (3). Recently, the United States Food and Drug Administration (FDA) approved pemigatinib, an orally bioavailable inhibitor of the fibroblast growth factor receptor (FGFR) 1/2/3, for patients with FGFR2 fusion or rearrangement (9–14% of patients) (4). However, these agents are effective only against cancers with specific genomic subsets (5). Therefore, other therapies targeting the remaining CCA phenotypes and genotypes are warranted.

During the past decade, immunotherapy, particularly immune checkpoint blockade (ICB), has achieved success in the treatment of multiple malignancies (6). Following the TOPAZ-1 trial, Durvalumab was approved by the FDA on September 2, 2022, to be used in combination with Gem+Cis for the treatment of adult patients with locally advanced or metastatic CCA (7). Unfortunately, the varied disease subsets, desmoplastic stroma, and the rich tumor microenvironment (TME) of CCA may contribute to immunotherapy resistance (8). Moreover, immunosurveillance and immune evasion between inflammatory cells and cancer cells in the TME also contribute to immunotherapy resistance (9). Therefore, understanding the natural development and progression of the immune TME will provide insight into immunotherapy for CCA. Recent research found that effector lymphocytes, such as CD8^+^ cytotoxic T lymphocytes (CD8^+^ T cells) more aggressively infiltrate the fibrous septa compared with infiltration of the tumor lobules in CCA (10). As a result, ICB therapy is largely ineffective in patients with CCA that have a characteristically reduced effector immune cell infiltration (i.e., “cold tumors”). Traditional chemotherapy or radiotherapy is thought to change the CCA immune microenvironment; some treatments exert immunosuppressive effects, while others promote immunostimulation (11–13), yet none of them are sufficient to remodulate an “immune-cold” TME to an “immune-hot”. Therefore, novel approaches and methodologies for CCA treatments are in demand.

The highly malignant nature of CCA is associated with complex and dynamic interactions between tumor cells, stromal cells and the extracellular environment (14). Due to its adhesive nature, CCA is associated with the presence of a large number of stromal cells (15). The stroma of CCA contains non-immune and immune cell types, as well as capillary networks including tumor-associated fibroblasts, tumor-associated endothelial cells and lymphocytes such as tumor-associated macrophages (TAMs), tumor-associated neutrophils and regulatory T lymphocytes (Tregs) (16). Stromal cells are recruited and activated by tumor cells and, in turn, deleteriously shape tumor behavior through the release of a wide variety of paracrine signals, including cellular/chemokines, growth factors, morphogenesis and proteases (17, 18).

Single-cell RNA sequencing (scRNA-seq) analysis has become a powerful tool for revealing cellular diversity and intercellular communication at single-cell resolution, through which different functional sub-clusters of immune cells are identified (19). scRNA-seq sequencing of the stroma yields key functional signatures of TME in CCA and is paving the way for immunotherapy and cancer-related fibroblast and extracellular matrix-directed therapies (20). scRNA-seq has shown that the vascular-associated fibroblast subclass is characterized by an enrichment of the microvascular system, combined with a high level of IL-6 secretion, and subsequent upregulation of the zeste 2 polycomb repressive complex 2 subunit enhancer induces significant epigenetic changes in CCA cells (21). This approach has greatly improved our understanding of tumor pathogenesis and facilitated the screening of potential tumor biomarkers and promising therapeutic targets. scRNA-seq has emerged as a powerful tool for studying complex cellular components in CCA tumor microenvironments.

Here, we review the recent advances in the role of the immune system in CCA, based on the crosstalk between the innate/adaptive immune system and the TME, mainly focusing on the opportunities and challenges of future immune-based therapy in CCA.

## 2 The role of the immune system in CCA

### 2.1 The innate immune system

**Figure 1:**
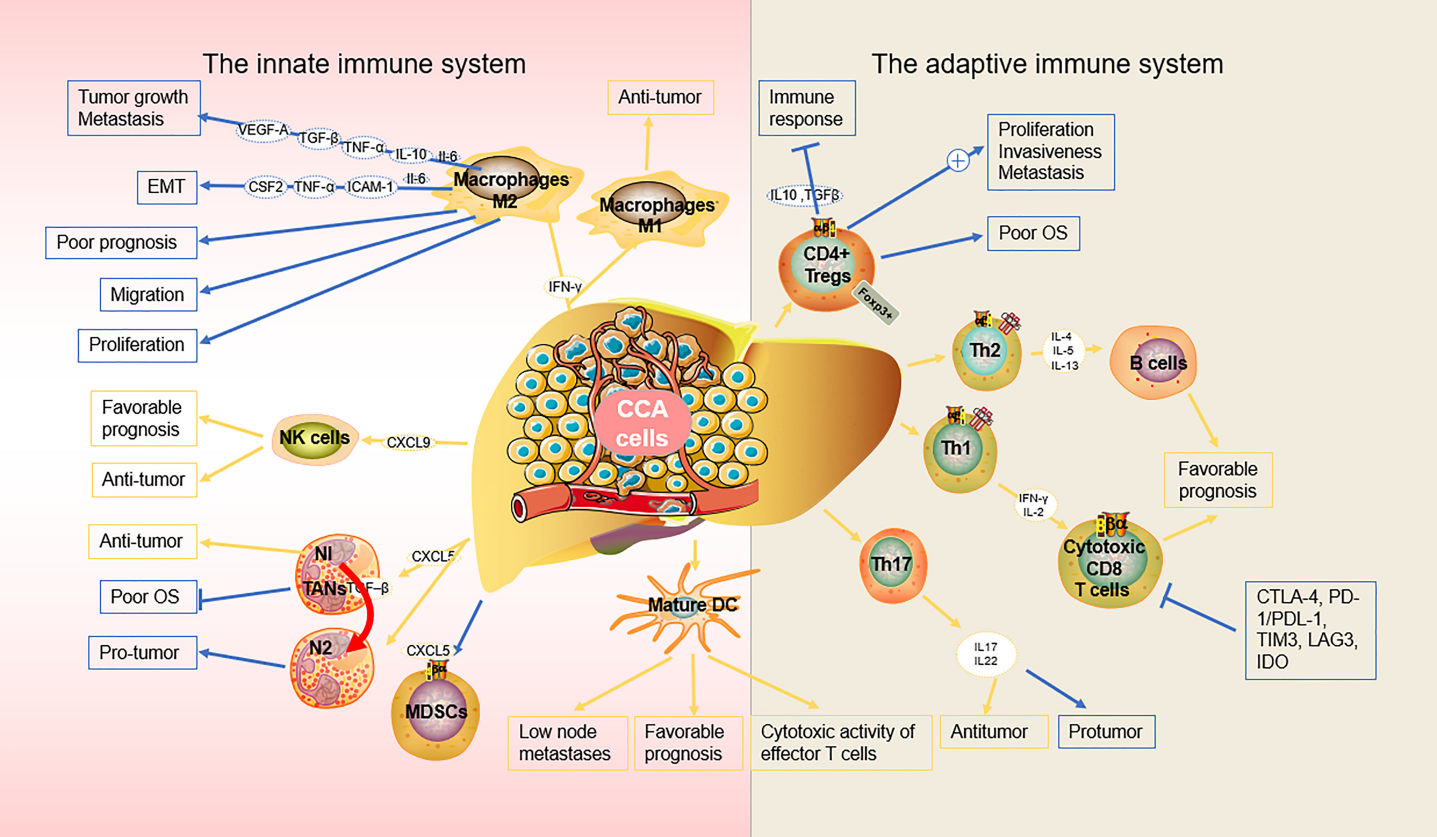
Biological role of the main immune components in the microenvironment of CCA.

Some immune cells and their associated cytokines act against tumor proliferation while others exert pro-tumor effects, indicating the complexity of the mechanisms underlying the immune response and the need for further studies to optimize immunotherapy (22) (**Table 1**) (23, 24) (**Figure 1**).

**Table 1:** Role of main immune components in microenvironment and potential targets.

Innate immune component	Type	Secret cytokines/ chemokines	Interaction with TME
Macrophages	M1	IL-1, IL-12, CXCL10, interferon (IFN)-γ, TNF-α and inducible nitric oxide synthase (iNOS), etc.	Immunostimulation
	M2	IL-4, IL-6, IL-10, IL-13, TNF-α, GM-CSF, ICAM-1, VEGF, EGF, CCL1, CCL3, CCL17, CCL22, CCL24, etc.	immunosuppressive
Kupffer cells	M1	NO, TNFα, IFNγ, IL-1β, IL-6, COX-2, etc.	Immunostimulation
	M2	IL-4, IL-10, IL-13, TGF-β.	Immunosuppressive
Natural killer cells		IFN-γ, TNF-α, GM-CSF, CCL3, CCL5, etc.	Immunostimulation
Natural killer T cells		perforin, Fas ligand, granzyme B, TNFα.	Immunostimulation
Dendritic cells		IL-12, CXCL9, CXCL10.	Immunostimulation/ immunosuppressive
Myeloid-derived suppressor cells		ARG1, iNOS, TGFβ, IL-10, COX2, IDO.	immunosuppressive
Tumor associated neutrophils	N1	INFγ, NETs, ICAM1, TNF-a, CXCL10, CCL7, CCL2, CCL3. etc.	Immunostimulation
	N2	IL-4, IL-8, IL-13, CCL2, CCL3, CCL4, CCL5, CCL8, CCL12, CCL17, CXCL1, CXCL2, CXCL8, CXCL16. etc.	immunosuppressive
Tumor-associated embryonic substance		VEGF, PGE2, TGFβand IL6 (15). Endothelial nitric oxide synthase (eNOS), arginase and IDO (16).	immunosuppressive
B lymphocytes	Bregs	IL-10, IL-35, TGFβ, Lymphotoxin.	immunosuppression
	B effector cells	IgG, IgM, IFN-y, ILs, TNFs, IFNs, Fas/FasL, TRAIL/Apo2L.	Immunostimulation
T lymphocytes	CTL cells	perforin, granzymes, and granulysin, Fas/FasL.	Immunostimulation
	Th Cells	IFNγ, IL-2.	Immunostimulation
	Tregs	TGF-β, IL-10, and IL-35.	immunosuppression

The innate immune response is the first line of defense against cancer cells in the TME and plays a critical role in both initiating antitumor immune responses and tumor progression (25). Improving current immunotherapeutic approaches depends on the understanding of the inhibitory and excitatory interactions that the innate immune system has with the CCA microenvironment. Here, we analyze recent discoveries in the role of various cell populations within the innate immune system in the TME, particularly in CCA, with a focus on macrophages, Kupffer cells (KCs), natural killer (NK) cells, natural killer T cells (NKTs), dendritic cells (DCs), myeloid-derived suppressor cells (MDSCs), and tumor-associated neutrophils (TANs).

#### 2.1.1 Macrophages

Macrophages are highly plastic cells capable of transforming into different phenotypes, including M1-like and M2-like macrophages, which are crucial for the function of the innate immune system. M1-like macrophages play a pivotal role in proinflammatory signaling, providing a positive feedback loop in the anti-tumor innate immune response (26), while M2-like macrophages are involved in the inhibition of inflammation, tissue repair and remodeling, angiogenesis, and tumor progression (27). CCA cells can produce a significant amount of interleukin-6 (IL-6) and transforming growth factor-beta (TGF-β) and induce macrophage polarization towards the M2-like phenotype *via* the IL6/STAT3 pathway (28). M2-like macrophages, also known asTAMs, facilitate tumor development and progression. In patients with iCCA, TAM-infiltrated hepatic macrophages were activated predominantly as M2-like subsets, which also infiltrate tumor lobules and construct a crosstalk bridge between the innate immune response and the TME (28). TAMs not only secrete cytokines, such as tumor necrosis factor-α (TNF-α), IL-6, granulocyte-macrophage colony-stimulating factor (GM-CSF), intercellular adhesion molecule 1 (ICAM-1), vascular endothelial growth factor (VEGF), and epidermal growth factor (EGF), but also various chemokines (CCL1, CCL 3, CCL 17, CCL 22, and CCL 24). Sun et al. reported that M2-TAMs promote iCCA cells *via* epithelial-mesenchymal transition (EMT), partially *via* releasing CSF2, TNF‐α, ICAM‐1, and IL‐6, subsequently activating the AKT3/PRAS40 signal transmission (29). Thanee et al. demonstrated that M2-TAMs correlate with CCA extrahepatic metastases, possibly *via* the EMT processes (30). They also found that the conditioned medium of M2-TAMs promoted CCA migration. Besides metastasis, M2-TAMs also correlate with the iCCA pathological grade and microvascular density (15, 28). Moreover, M2-like macrophages can also attract immune cells that are mostly immunosuppressive, such as TANs, MDSCs, or Tregs by secreting IL4, IL8, IL10, CCL2, CCL22, and CCL17 (31). Furthermore, macrophage‐derived Wnt ligands, including Wnt3a and Wnt7b, promote CCA cell proliferation *via* the canonical Wnt pathway (32, 33).

CCA is a lethal tumor that possesses a large number of immunosuppressive bone marrow cells in the TME, including TAMs and bone marrow-derived suppressor cells (34). CCA is characterized by a prominent desmoplastic TME composed of various cell types that support and promote tumor progression (e.g., infiltrating immune cells and cancer-associated fibroblasts, CAFs) and extracellular components (35). CCA cells and other components of TME, direct macrophages to tumors by releasing multiple factors including MCP-1/CCL2, CSF-1, and VEGF-A (36). The recruitment and differentiation of macrophages into TAMs are driven by specific subpopulations of CCA cells mediated by IL-13, IL-34, and osteoactivin (15). In hilar CCA, the presence of some macrophages expressing the angiopoietin receptor TIE2 (TEMs) is related to a favorable prognosis (37). However, if a high number of these immunosuppressive TAMs infiltrate the tumor, patients with CCA display unfavorable disease-free survival and poor prognosis (28, 29). Fortunately, this allows for the use of TAMs as therapeutic targets. For example, the high rate of tumor infiltration with TAMs, as observed in certain cancers like eCCA, demonstrates the potential efficacy of TAM-targeted, synergistic immunotherapies, thereby enabling the development of optimized treatment (38). Xu et al. showed that MMP19 and SIRPα could predict the ICB response in iCCAs (37). Although increased expression of MMP19 and SIRPα was predictive of poor prognosis for iCCAs without postoperative immunotherapy, patients with increased expression of SIRPα were more sensitive to immunotherapy, while patients with increased expression of MMP19 were not sensitive to immunotherapy (39).

Due to immunosuppression and the limiting effects of ICB, targeting MDSCs and blocking MDSC recruitment may be an attractive therapeutic opportunity (40). However, information regarding the phenotypes of MDSCs in the CCA microenvironment remains limited. More importantly, the reason for MDSC accumulation and activation in CCA compared with that in the surrounding healthy tissues in specific patients remains poorly understood. Therefore, the above question needs to be addressed so that targeting the MDSCs can become a feasible therapeutic approach to CCA.

Kupffer cells (KCs) are a subset of macrophages that combat metastasis through the recruitment and activation of NK cells, NKT cells, DCs, and cytotoxic molecules such as TNF-α (41). Conversely, KCs promote cholangiocyte proliferation and carcinogenesis by expressing increased levels of TNF-α near the iCCA lesions (42). Therefore, CCA cells can resist attacks from immune cells through an up-regulated production of inflammatory cytokines and chemokines, contributing to TME remodeling (43).

#### 2.1.2 NK cells

NK cells are a type of innate lymphoid cells that provide host defense against tumors, in their microenvironment, through their potent cytolytic function. Particularly in CCA, NK cells are the predominant immune cells (44). It has also been demonstrated that biliary epithelium can present lipid antigens to NK cells and activate them *via* the non-polymorphic major histocompatibility complex (MHC) homolog CD1d (45). NK cells are abundant in the liver where they prevent cancer cells from invading the liver and induce cancer cell death through a variety of cytotoxic pathways, including particle-mediated, FasL-mediated, and TNF-mediated apoptosis (46). Here, we discuss the role of NK cells in the innate immune response against cancer and the new therapeutic approaches utilizing NK cells in CCA. In iCCA, increased expression of the endogenous CXCL9 closely correlates with prolonged survival by regulating tumor-infiltrating NK cells and (47) augmenting anti-tumor immune surveillance (46). Gentilini et al. (48) found that transfusions of *in vitro* amplified human NK cells (SMT01) into nude mice carrying HuCCT-1 tumors significantly inhibited the growth of CCA. *In vitro*-activated NK cells augment the cytotoxic efficacy of cetuximab against human CCA cell lines (47). In a nude mice xenograft model of CCA, Jung et al. demonstrated that NK cells are effective against CCA through their characteristic cytolytic activity (49). At the same time, activation of aberrant anti-apoptotic cascades in CCA enhances NK cell-mediated resistance to apoptosis (50).

Further investigation of NK cells-based immunotherapy can help determine cancer therapeutics for CCA.

#### 2.1.3 DCs

DCs are innate immune cells primarily responsible for recognizing and responding to foreign pathogen-associated signals, presenting antigens for activating naïve T cells, and shaping the acute immune response (51). Immature DCs have a high capacity for endocytosis of lactose antigens and become mature in response to various stimuli by binding to pattern recognition receptors, characterized by costimulation (CD40, CD80, and CD86) and increased surface expression of MHC molecules. Mature DCs strongly promote immune responses (52). Previous studies have shown that treatment with CD40 agonists in combination with anti-PD1 antibodies significantly increases the number of DC cells in ICC tissue and limits tumor growth (53). DCs are also critical for the induction of both an antitumor response (54, 55) and an immunosuppressive state (56) in the TME.

In CCA, mature DCs surrounded by CD4^+^ and CD8^+^ cells are observed at the cancer periphery, indicating that DCs might function as a bridge between the innate and the adaptive immune response, which may reflect immune exclusion in the TME. Among patients with CCA, those that exhibit mature CD83^+^ DCs had a better prognosis and lower incidence of lymph node metastases than those that are CD83^−^ (57). In CCA, infiltration of mature CD83+ dc was associated with the accumulation of CD4+/CD8+ T cells in the peritumoral region. the presence of CD83+ dc was also associated with improved patient prognosis (58). CCA cells and their microenvironment secrete immunosuppressive cytokines, such as transforming growth factor-β (TGF-β) and IL-10, which inhibit the function of DCs, resulting in the reduced antitumor activity of T cells (59). Panya et al. developed self-differentiated monocyte-derived DCs (SD-DCs) that could express a cAMP-dependent protein kinase type-1 alpha regulatory subunit. These artificial DCs could significantly enhance the cytotoxic activity of effector T cells, inducing a stronger adaptive immune response against CCA (60). Correspondingly, the reduction of classical DCs and TNF-α-producing proinflammatory DCs results in a defective DC-mediated immune response in patients with iCCA (61). Sadeghlar et al. (62). found that active DCs induced Th1 cytokine expression in effector cells, proliferation, and tumor-specific cytotoxicity against CCA.

Due to the dual role of anti- and pro-immune responses of DCs, and the complicated involvement of DCs in the TME, their applications in cancer immunotherapy in CCA still have a long way to go.

#### 2.1.4 TANs

TAN cells represent a subset of neutrophils that infiltrate tumors and play a complex role in the innate immune system (63). Through the release of different cytokines or chemokines, TANs can either present an anti-tumorigenic phenotype mediated by T cells called the N1-phenotype, or facilitate a pro-tumorigenic phenotype called the N2-phenotype for tumor promotion in different TMEs. Fridlender et al. suggested that TGF-β is the key cytokine that defines the TAN phenotype and skews differentiation from the N1 anti-tumorigenic phenotype to the N2 pro-tumorigenic phenotype (64).

In CCA, neutrophil recruitment is mainly induced by CXCL5 *via* PI3K-Akt and ERK1/2-MAPK pathways, thereby promoting iCCA growth and metastasis (65). A positive correlation between the number of TANs in patients with CCA has been reported; TAN is a key regulator of inflammation and immune status (66). Previous studies have also reported an association between TANs and poor overall survival in eCCA (66) and iCCA (67), and shorter disease-free survival (68). Though there are various advances in the field of TANs-based cancer therapy, such as the targeting and exploitation of N1 TANs to enhance immunotherapy for cancer treatment, data on the immunosuppressive effects of TANs remain inconclusive (69). Their relationship with the rest of the TME as well as the pro- and anti-carcinogenic properties of TAN subtypes require further investigation prior to clinical application (69). The exact property of TANs in the CCA microenvironment remains controversial and understanding the role of TANs as pro- or anti-tumor will help develop strategies for cancer therapy (70).

### 2.2 The adaptive immune system and crosstalk with TME

#### 2.2.1 Tumor-infiltrating T lymphocytes

The adaptive immune system encompasses humoral and cell-mediated responses. It is antigen specific, in terms of recruiting certain cells under specific conditions for the targeted destruction of cancer cells. The TME recruits a variety of tumor-infiltrating lymphocytes (TILs) from the vasculature, most of which are white blood cells. In the adaptive immune system, B cells and T cells constitute the vast majority of TILs (71). This includes CD8^+^ cytotoxic T lymphocytes, CD4^+^ T helper lymphocytes, and CD4^+^ T regulatory cells, all of which play diverse roles in the function of the adaptive immune system in the TME. During CCA initiation, development, and metastasis, local imbalances of T cell subsets in the adaptive immune system and CCA microenvironment have attracted interest (48, 66, 72, 73). In one cohort of patients with CCA, a higher number of infiltrating total lymphocytes, B cells, CD4^+^, and CD8^+^ T cells was described as a favorable prognosis marker (74). Systematic evaluation of T cell subsets may be key to the development of effective immunotherapies.

##### 2.2.1.1 CD8^+^ cytotoxic T lymphocytes

CD8^+^ T cells are robust immune cells that play a central role in the adaptive immune response, controlling cancer growth within the TME (75). More than half of resected CCAs are positive for CD8^+^ TILs, of which, 30% are positive for Granzyme B, indicating an activated and cytotoxic phenotype (68). Xia et al. (44) found that Granzyme B+ CD8^+^ effector T cells were significantly associated with overall survival in iCCA and dCCA. CD8^+^ T cells directly destroy tumor cells by releasing proteins such as perforin and granulozyme, and indirectly induce apoptosis by expressing FasL or secreting TNF-α attached to target cell surface receptors (76). Notably, Asahi et al. demonstrated that the accumulation of CD8^+^ T cells around the outer border of the tumor also positively correlates with an improved prognosis in postoperative patients with CCA (77). However, in another CCA cohort, Gu demonstrated that an increased number of CD8^+^ T cells are preferentially present at the tumor invasive front than in the intratumor area, suggesting an immunosuppressive microenvironment inside of the tumor (67). A recent study identified that intratumoral CD8^+^ T cell infiltration can even up-regulate PD-L1 expression in affected cancer cells, leading to adaptive immune resistance and tolerance, in CCA (78). CD8^+^ T cells, which are part of the immune cells in the TME, are common in iCCA and significantly affect the occurrence of iCCA. CD8^+^ T cell infiltration is associated with better survival and lower recurrence (79). Meanwhile, the potential use of CD8^+^ T cells as a prognostic marker for CCA has also been highlighted, as a significant increase in CD8^+^ T cell density is seen in lymphoepithelial subtypes of Epstein-Barr virus-associated CCA and is significantly associated with favorable prognosis for iCCA (80).

##### 2.2.1.2 CD4^+^ T helper lymphocytes

CCA cells induce apoptosis of CD4^+^ and CD8^+^ T cells through the Fas/FasL pathway in tumor cells (81). CD4^+^ T helper lymphocytes (Th cells) play a central role in the different stages of adaptive immune responses. In the TME of CCA, Th cells mainly accumulate at the tumor’s outer border margin (82) where they are essential in helping B cells produce antibodies to inhibit tumor cell proliferation, while CD8^+^ T cells phagocytose tumor cells (83). Similar to many of the other cell types previously discussed, Th cells have multiple heterogeneous subtypes that can confer both protumor and antitumor effects. Based on specific cytokine secretions, Th cells can differentiate into Th1, Th2, and Th17 cells. Th2, Th1, and Th17 cells produce IL-22, and this receptor activation induces proliferative and antiapoptotic signals through signal transducers and transcriptional activators (84). Generally, Th1 cells mainly produce interferon- γ (IFN-γ) and IL-2 cytokines, activate CD8^+^ T cells, and initiate antitumor response (85).

Th2 cells are primarily responsible for IL-4, IL-5, and IL-13 production, which is an integral part of the B cell-mediated immune response (86). However, the Th17-derived cytokines, IL-17 and IL-22, show both protumor and antitumor behaviors. These range from facilitating an inflammatory response to controlling the activation of myeloid cells and other T cells (87).

In a patient with metastatic CCA, Tran et al. successfully inhibited lesion progression and prolonged stabilization of disease by using adoptive transferring of mutation-specific Th cells (88). TILs from patients with metastatic CCA contain CD4^+^ Th1 cells and recognizable mutations in cancer-expressed erbb2 interacting proteins (80). This suggests that the Th cell response can be harnessed to mediate regression of advanced CCA (88). Undoubtedly, Th cell-based therapies, focused on manipulating the TME, will play an important role in CCA control and patient management in the future (89).

##### 2.2.1.3 CD4^+^ T regulatory cells

CD4^+^ Tregs differentiation depends on the expression of Forkhead box P3 (FoxP3), an interaction critical towards effective adaptive immune responses (90). Ma et al. identified the role of FoxP3 in tumor malignancy, as the downregulation of FoxP3 inhibits proliferation, invasiveness, and metastasis of tumor cells, reduces IL-10 and TGF-β signaling, and blocks immune escape in CCA (91). Similarly, CCA cells activate natural Treg-like CD4^+^CD25^−^ cells by increasing TGF-β and IL-2, thereby compromising the immune response (92). Another study confirmed that numerous Treg populations in the TME are associated with lymphatic metastasis and worse overall survival in eCCA patients, even with surgical resection (66, 93).

Therefore, a deep understanding of the pathways and mechanisms leading to clonal enrichment of infiltrating Tregs and exhausted CD8^+^ T cells in CCA will provide better strategies to orchestrate the immune system to eradicate cancers.

## 3 Future direction and outlook for CCA management

**Figure 2:**
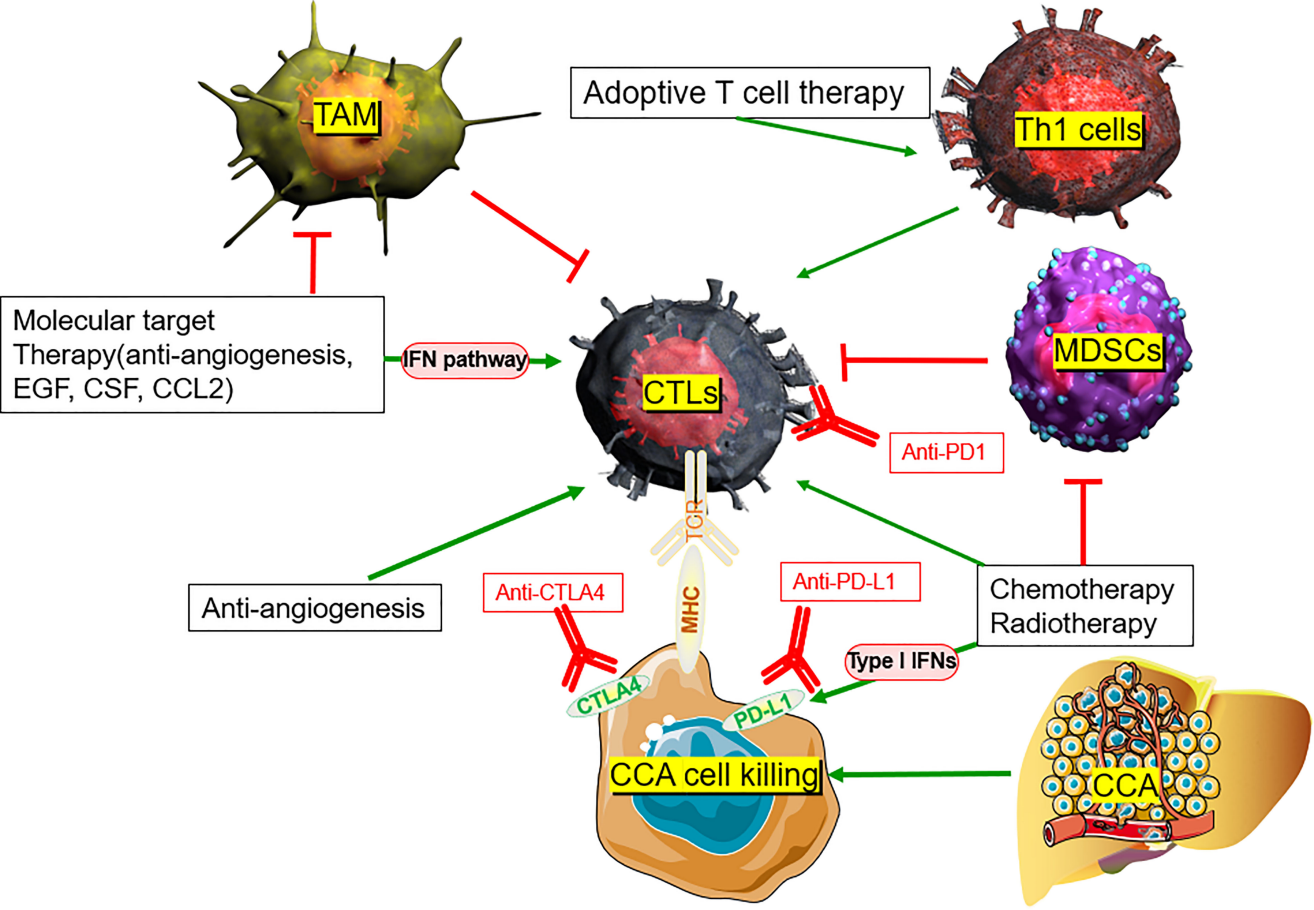
TME rationale of combination of ICB with other potential therapeutic strategies in CCA.

There is no systemic clinical management available for patients with advanced CCA and disease progression following the current standard treatment, i.e., chemotherapy with Gem+Cis. Additionally, the defensive functions of immune cells are suppressed in CCA. Cytotoxic T cells and NK cells have poor tumoral infiltration, and Tregs accumulate intratumorally. Overexpression of PD-1, CTLA-4, and GITR also have negative effects on TILs, bolstering immunosuppression of the tumor. Aside from ICB therapy, other potential treatments for CCA, such as peptide and DC-based vaccines and adoptive T cell therapies, are currently being evaluated. Both stimulating or blocking immune cells through immunotherapy in the TME of CCA and using these vaccines in conjunction with other conventional therapies show great potential (**Table 2**) (88, 94–107) (**Figure 2**).

**Table 2:** Current immunotherapeutic strategies to treat CCA.

Immunotherapy	Mode of action	Application details
Immune checkpoint blockade	Inhibit immune checkpoints such as PD-1, PD-L1, CTLA-4, TIM3, LAG3, IDO and others.	Anti-PDL1: (atezolizumab, NCT03201458), anti-LAG3 (Sym022, NCT03489369).
Cell therapy	Transfer of tumor-specific immune cells into CCA patients. Patient-derived immune cells are modified ex vivo and retransferred into the donor.	Th1 cells (78), Activated T-cell transfer (84), CART (85), allogenic gammadelta T cell (86), TAA-specific CTL (87), Allogeneic NK Cell (NCT03358849), T Cells Modified With Chimeric Anti-CEA Immunoglobulin-T Cell Receptors (IgTCR) (NCT00004178).
Vaccines	Tumor-associated antigens are targeted to overcome immune tolerance.	Antigenic peptides: WT1 (87), MUC1 (88), MUC5AC (87), CEA (87), CEA RNA-pulsed DC cancer vaccine (NCT00004604), PPV (89), Dendritic cell-based vaccines (90), TRICOM-CEA(6D) (NCT00027534), TRICOM-CEA(6D) (NCT00027534), .
Oncolytic virus	Enhances cytotoxicity and inhibits tumor growth	adenovirus (91, 92), vaccinia virus (93), measles vaccine virus (MeV) (94), herpes simplex virus (95, 96),
Combinations with other immunotherapies.	Inhibit multiple immune checkpoints such as PD-1, PD-L1, CTLA-4, TIM3, LAG3, IDO and others	nivolumab plus ipilimumab (anti-PD-1 plus anti-CTLA-4) (NCT02923934, NCT03101566) (97), XmAb20717 (anti-PD-1 plus anti-CTLA-4, NCT03517488), LY3434172 (anti-PD-1 plus anti-PD-L1, NCT03936959), Durvalumab plus Tremelimumab (anti-PD-L1 plus anti-CTLA-4, NCT02821754), LY3321367 plus LY3300054 (anti-TIM3 plus anti-PD-L1, NCT03099109), REGN3767 plus REGN2810 (Anti-LAG-3 plus Anti-PD1, NCT03005782), Lirilumab plus an Nivolumab (Anti-KIR plus Anti-PD1, NCT01714739), Durvalumab plus SNDX-6352 (anti-PD-L1 plus CSF1R, NCT04301778), INT230-6 plus anti-PD-1 and anti-CTLA-4 (NCT03058289), recombinant fowlpox-CEA(6D)/TRICOM plus sargramostim (vaccine plus GM-CSF, NCT00028496), XmAb22841(anti-CTLA-4 plus anti-LAG-3,NCT03849469), XmAb22841 plus Pembrolizumab (anti-PD-1 plus anti-CTLA-4 plus anti-LAG-3, NCT03849469) , NK Cell plus Pembrolizumab(anti-PD-1)( NCT03937895).

### 3.1 Immunotherapy

The rapidly growing efforts for improving immunotherapy have partly uncovered the underlying immune landscape of CCA and paved the way for immune-oriented clinical trials. However, the development of immunotherapy in this heterogenous and relatively rare malignancy is consistently challenging.

#### 3.1.1 Cell therapy for CCA

Adoptive T cell therapy is another potential treatment for CCA that uses cytotoxic T lymphocytes, occasionally from a donor, to more effectively infiltrate cold or immunosuppressive TMEs (108). Tran et al. transferred mutation-specific Th1 cells with somatic non-synonymous mutation-specific antigen-presenting cells to a patient with metastatic CCA, resulting in a positive immune response and a 30% reduction in tumor size (88). In a larger cohort, the median progression-free survival (PFS) and overall survival were significantly improved in 36 postoperative patients with iCCA receiving an adjuvant adoptive transfer of T cells plus a DC vaccine (94). Chimeric antigen receptor T (CAR-T) cell therapy is another innovative strategy for managing CCA. A phase I study of 11 patients with human epidermal growth factor receptor 2 (HER2)-positive advanced CCA (n = 9) treated with HER2-targeted CAR-T therapy demonstrated disease control in 4/9 patients with CCA, which included a PR that lasted 4.5 months (109). Likewise, Feng et al. successfully infused CAR-T cells targeting epidermal growth factor receptor (EGFR) and CD133 into a patient with metastatic CCA and achieved small increases in overall survival and cancer cell death, especially when combined with other therapies (95). Phanthapho et al. demonstrated that A20-2G and A20-4G CAR-T cells targeting integrin αvβ6 could effectively kill CCA cell lines, indicating their potential for CCA treatment. Allogenic gamma delta T cells, another potential immunotherapy, were used in a patient with CCA after liver transplantation and had similar increases in overall survival while also boosting peripheral immune function and reducing metastasis (96). In a 30-year-old male diagnosed with recurrent mediastinal lymph node metastasis after liver transplantation for stage IV CCA, the size and activity of the lymph nodes significantly decreased after adoptive γδ T cell transplantation (44).

However, larger cohorts of clinical trials are urgently needed to verify the toxicity, safety, and efficacy of adoptive T-cell therapy for CCA.

#### 3.1.2 Vaccine therapy for CCA

Three tumor antigens, such as CD247, FCGR1A, and Trap, have been identified in CCA and are associated with a good prognosis and antigen-presenting cell infiltration. They are also potential antigens for the development of CCA mRNA vaccines (110). Several vaccine-based strategies, including carcinoembryonic antigen RNA-pulsed DCs and immunogenic peptides plus gemcitabine, have been developed for CCA treatment, singly or in combination (111). Antigens are expressed in most tumors, referred to as tumor-associated antigens (TAA), which may become targets of antigen-specific T cell responses, leading to tumor rejection. Vaccines function as tumor rejection antigens to stimulate the host’s adaptive immune response in the TME. Using these techniques, various applications are undergoing active clinical evaluation in CCA (112). The safety, toxicity, tolerance, and anti-tumor efficacy of peptide vaccines, such as Wilms tumor 1 (WT1) peptide vaccine (97, 113), mucin 1 (MUC1) peptide vaccine (98), mucin 5AC (MUC5AC) peptide vaccine (97), and carcinoembryonic antigen (CEA) peptide vaccine (97), were verified in phase I clinical trials against advanced biliary tract cancers. Furthermore, the feasibility of a personalized peptide vaccination (PPV) was evaluated in a case report (93) and a phase II clinical trial, which indicated a significant positive effect on the immune response with almost nonexistent toxicity (99). DC-based vaccines were designed due to their characteristic activation of naïve T cells, which, in turn, will bolster acute antitumor responses (51, 114). *In vitro* experiments demonstrated that when DCs were combined with either specific tumor lysates (115) or messenger RNA (100), the resulting induction of T lymphocyte proliferation increases anti-tumor immune infiltration to target CCA cells. The combination of a DC-pulsed vaccine with *ex vivo* activated T cells appears promising for the treatment of biliary tract carcinoma (116). However, this conclusion lacks *in vivo* data and DC-based vaccines used in CCA have a long way to go before they replace current therapies (117). In a recent study by Pan et al., a CTLA4-PD-L1 chimeric protein vaccine (protein vaccine) was constructed, which may function as a therapeutic and preventive cancer vaccine in TAA-induced iCCA rat models (118). Hochnadel et al. showed that in the CCA environment, the LmAIO strain can induce Th1 immune response against tumor antigens, thus, promoting the destruction of tolerance and epitope diffusion of autoantigen (119). As a result, phase II, or III, clinical trials are urgently needed to verify patient tolerance and anti-tumor efficacy of peptide vaccines.

#### 3.1.3 Oncolytic virus therapy for CCA

The use of an oncolytic virus is another gene therapy that is useful against CCA, specifically targeting and killing infected tumor cells without affecting healthy cells. Recently, some virus-based therapies such as adenovirus (101, 102), vaccinia virus (103), measles vaccine virus (MeV) (104), and herpes simplex virus (105, 106) have been tested as a single modality or in combination in preclinical settings, exhibiting promising results (120). Specifically, conditionally replicative adenoviruses (CRAds) (101) and recombinant adenovirus (Adp27-jab-d), expressing p27-jab-d (102), effectively enhanced cytotoxicity and inhibited tumor growth in both *in vitro* and *in vivo* CCA. Adenovirus AxdAdB-3 reduced subcutaneous GBC tumor growth in nude mice compared with placebo, while the addition of 5-fluorouracil to viral treatment resulted in complete tumor regression in nearly 50% of treated mice (121). Oncolytic vaccinia virus demonstrated pan-cancer-specific lytic potency in different human and mouse CCA cell lines, showing the highest virulence among several comparable recombinant viruses, including vSC20, vSC65, vAng1, vTRAIL, and WT (122).

GLV-1h68, a replication-competent lysogenic vaccinia virus, efficiently infected, replicated, and lysed three different CCA cell lines in culture (123). This armed MeV vector (MeV P-SCD) could efficiently replicate in three different human CCA cell lines, leading to the expression of viral-encoded proteins and severe cell death in two of the three cell lines studied (104). Zhu et al. (101) reported the construction of three new CRAds agents that effectively target CCA cells, induce strong cytotoxicity *in vitro*, and inhibit tumor growth in mouse xenograft models *in vivo*. However, the major limitations of adenovirus-based therapy to clinical application are its insufficient infectivity and specificity towards tumor tissues, halting significant progress using this treatment. Despite these issues, other treatments, such as cancer-favoring oncolytic vaccinia virus (CVV) therapies, may have more promising results for uncured CCA patients (103). Slow growth and complete lysis within CCA tumor blocks in CVV-treated nude mice in a xenograft model demonstrate the therapeutic effect of CVV on CCA tumors (103).

#### 3.1.4 ICB for CCA

Immune checkpoint molecules, such as CTLA-4 (124), PD-1/PD-L1 (125), T cell immunoglobulin, mucin domain-containing 3 (TIM3) (126), lymphocyte activation gene 3 (LAG3) (127), and IDO (128) are currently being assessed for their immune inhibitory potential in the TME. PD-1/PD-L1 and CTLA-4 have attracted attention in clinical trials. Sabbatino et al. and Gani et al. reported that PD-L1 expression by CCA cells provides tumor cells with an efficient immune escape mechanism, leading to poor tumor differentiation, higher malignant tumor stage, higher levels of apoptotic CD8^+^ TILs, and eventually poor patient survival (10, 129). Meanwhile, Lu et al. (130) found that PD1/PD-L1 signaling was activated in a large group of iCCA tumor tissues and that elevated PD1/PD-L1 signaling was positively correlated with a malignant phenotype such as lymph node infiltration and high TNM stage. Nakamura et al. (131) found that the subgroup with the worst prognosis was characterized by an elevated expression of immune checkpoint molecules such as PD-L1, based on genomic and transcriptomic status. In addition, PD-L1 expression rates range from 17.7 to 72.2% in different ICC cohorts, with T cell infiltration present in most ICC samples (132). Zhou et al. observed that CTLA-4 is over-expressed on the surface of TILs in CCA (83). Furthermore, higher expression of both CTLA-4 at the tumor-host interface of iCCA correlates with tumor recurrence and chemo-resistance (74). Antagonistic targeting of CTLA-4 enhances effector T cell proliferation, which was observed in the *ex vivo* stimulation of TILs derived from CCA (83). These findings suggest that these molecules are ideal targets for therapeutic stimulation of immune cells in several clinical trials on CCA management (**Table 3**).

**Table 3:** Immune-related biomarkers in microenvironment and clinical molecular target in CCA.

biomarkers	Site	Biological significance
CTLA-4	Treg cells.	Immune checkpoint molecules: Surface protein binding to CD80 on antigen-presenting cells to inhibit cytotoxic cells.
PD-1	Immune cells.	Immune checkpoint molecules: surface protein binding to PD-L1 on cancer cells leading to immune escape.
PD-L1	Tumor cells, immune cells.	Immune checkpoint molecules: surface protein binding to PD-1 on immune cells leading to immune escape.
LAG3	Immune cells.	Immune checkpoint molecules: reduce T cell proliferation and cytokine secretion.
TIM-3	T cells, Treg cells, macrophages, DCs.	plays a key role in inhibiting Th1 responses and the expression of cytokines such as TNF and INF-γ
IDO	DCs, macrophages, and fibroblasts	suppression of CTL cells and NK cells as well as increased activity of Tregs and MDSCs.
ICOS	T cells.	induction and regulation of Th1, Th2, and Th17 immunity.
VEGF/VEGFR	Endothelial cells.	Activation of immunity by increasing the expression of endothelial cell adhesion molecules that directly interact with immune cells for antigen recognition, rolling, adhesion and extravasation during immune responses. Inhibiting the maturation of DCs, which suppresses immune activation.
PDGF	Tumor cells.	PDGF-D released by tumoral ducts attracts and activates liver fibroblasts to secrete VEGF-C/VEGF-A leading to tumor-associated lymphangiogenesis and lymphatic invasion.
TGF-β	Tumor stroma.	Pro-fibrogenic cytokine.
CSF1/CSF1R	Monocytes, Mo-MDSCs, macrophages.	supports differentiation and survival of TAMs, tumor promoting and immune suppressive. Critical drivers of immune escape in the TME include TAMs and MDSCs.
TIE2	TAM and Monocyte.	Distinct paracrine inducers of angiogenesis, convert T cells into Tregs and suppress tumor-specific immune responses.
FGFR	Tumor cells.	FGFR fusions result in constitutive tyrosine kinase activity, which in turn led to downstream signaling pathways activation, PI3K activation enhances immune suppressor and pro-angiogenic potentials of TAMs.
EGFR	Tumor cells.	Remodels the TME to trigger immune escape by upregulation of PD-1, PD-L1, CTLA-4, and multiple tumor-promoting inflammatory cytokines.
Mesothelin	Tumor cells.	It is an attractive target for cancer immunotherapy because its normal expression is limited to mesothelial cells, which are dispensable.
Flt3/Flt3L	Hematopoietic stem cells / progenitor cells	increases the number of immune cells (lymphocytes (B cells and T cells)) by activating the hematopoietic progenitors. stimulates the development of NK cells and DC cells.
IFN‐γ	Activated T cells and NK cells	critical to both innate and adaptive immunity, and functions as the primary activator of macrophages, NK cells, a master checkpoint regulator for many cytokines.

Although most clinical trials investigating ICB in advanced CCA have failed to produce significant clinical outcomes, modest but real responses in all subtypes and meaningful disease control were recorded in CCA treated with ICB, with objective response rates (ORRs) ranging from 5 to 20% (133). Moreover, the advancement in biomarker-based ICB has shed light on the complex biological heterogeneity within these tumors. Approximately 6% of patients with eCCA have overexpression of PD-1 and PD-L1 in the tumor area, which is linked to increased tumor progression and metastases, particularly when accompanied by low CD3^+^ or CD8^+^ T cell infiltration (134). Lu et al. reported that in a cohort of Hepatitis B Virus (HBV) infected patients with CCA, PD-1 T cells could be used as biomarkers to predict prognosis and assess the efficiency of ICB therapy (73). Furthermore, Montal et al. identified that the upregulation of PD-1/PD-L1 is associated with a favorable response to ICB in eCCA (135). Fontugne et al. (136) showed that PD-L1 expression in tumor tissue from patients with iCCA is a biomarker to predict the efficacy of PD-1 inhibitor therapy, while Ye et al. (73) reported a negative correlation between CD8+ T lymphocytes and PD-L1 expression in iCCA, with PD-L1 being a negative regulator of T lymphocytes.

Lee et al. (137) found that in Gem+Cis refractory CCA and PD-L1 positive patients, pembrolizumab monotherapy showed durable anti-cancer effects in approximately 10% of the study population with a manageable overall safety profile. In addition, upregulation of PD-L1 may promote ICC cell invasion and migration (138).

Although recent phase I clinical trials have shown limited overall efficacy of ICB in CCA, there have been promising aspects of this therapy on a substantial portion of CCA tumors (139). In a prospectively planned retrospective analysis of patients enrolled in a multicohort clinical trial (KEYNOTE-158, NCT02628067), pembrolizumab showed limited antitumor activity in 104 previously treated patients with advanced CCA. ORR was 5.8%, complete response (CR) was achieved in no patients, and partial response (PR) was achieved in six patients. KEYNOTE-028 (NCT02054806) is a non-randomized, phase Ib trial in which pembrolizumab showed limited antitumor activity in 23 previously treated patients with advanced CCA. ORR was 13.0%, CR was achieved in no patients, and PR was achieved in three patients. In this report of patients with advanced CCA enrolled in two clinical studies, patients with a typical response to pembrolizumab monotherapy had a persistent response, all lasting ≥ 6 months. A response duration of ≥ 24 months was estimated in at least half of patients with a manageable safety profile (140). Further research can help clinicians to accurately identify patients who will maximally benefit from ICB cell therapy for CCA.

Immunotherapy used as a solitary treatment is insufficient to treat solid tumor cancers such as CCA. Vaccines, upregulation of selected cytokines, agonistic activation of costimulatory receptors, oncolytic viruses, and other cellular therapies can encourage tumor inhibition, while their combination with ICB provides an immunostimulatory effect. As a result, there is a significant benefit of combination therapies using molecular treatments targeting immunotolerant cells.

### 3.2 Combination therapy

#### 3.2.1 Combinations with other immunotherapies for CCA

Combinations of two or more ICBs make it possible to enhance the function of immunotherapy across a wide spectrum of malignancies (107). Some in-progress clinical trials further explore the effects of the anti-PD-1/PD-L1 and anti-CTLA4 combination (**Table 2**). The above clinical data have indicated that compared with monotherapy, dual or even triple ICB synergy presents the potential to improve outcomes. However, given the limited data, further Phase II/III clinical trials are necessary before combination ICB therapy sees widespread use. In addition, the combination of TIM-3, LAG-3, KIR, or several other novel immune checkpoint inhibitors (ICI) also shows potentially effective results (**Tables 2**, **3**).

There is growing evidence that ICIs are effective, and these inhibitors are now FDA-approved for a small subset of patients with CCA, including those with tumors with deficient mismatch-repair (dMMR) or microsatellite instability (MSI-H) and high tumor mutational burden (TMB; ≥10 mutations/million bases) in treatment-refractory settings (141).

However, there are no convincing data on co-inhibitory checkpoint therapy for CCA. Further studies are desperately needed to further assess the safety and efficacy of these combination therapies in advanced CCA.

#### 3.2.2 Immunotherapy plus chemotherapy for CCA

Sabbatino et al. showed that PD-1-expressing lymphocytes can only properly infiltrate the fibrous septa, while tumor lobule lymphocyte infiltration is almost nonexistent in iCCA (i.e., cold tumors) (10). This indicates that even if PD-1 blockade could partly overcome the tumor’s immune tolerance, the lack of cytotoxic T lymphocyte (CTL) activity within the tumor remains a barrier to achieving an effective anti-immune response against iCCA. The decreased immune infiltration of iCCA tumors potentially explains the poor efficacy of PD-1 monotherapy.

However, recent evidence suggests that standard chemotherapy (Gem+Cis) may stimulate beneficial changes in both the tumor and the immune system in the TME, thereby inhibiting immunosuppressive cells while increasing immunogenicity. Gem+Cis combination enhances the immunogenicity and antigenicity of different tumors, such as lung cancer, kidney cancer, colon cancer, breast cancer, and prostate cancer, by upregulating the expression of human leukocyte antigen (HLA) class I molecules on cancer cells (142). In CCA, Koido et al. demonstrated that gemcitabine induces expression of WT1, PD-L1, and calreticulin mRNA of the cancer cells (143). Recently, Sawasdee et al. demonstrated that gemcitabine also upregulates the immune functions of CTLs, resulting in a > 250% increase in their tumor cytotoxic effects when compared with untreated CCA cells *in vitro* (144). A Phase II clinical trial (NCT03951597) is currently testing gemcitabine in conjunction with PD-1 antibody ICB (JS001), while other clinical trials are investigating nivolumab or pembrolizumab plus Gem+Cis (145). In the TOPAZ-1 trial, phase III randomized, double-blind, placebo-controlled Gem+Cis combination plus durvalumab (GC-D) was compared with Gem+Cis plus placebo (146). Based on the recently announced increase in survival with GC-D compared with Gem+Cis alone in TOPAZ-1 trials, ICI-based systemic therapy is expected to be a new first-line treatment option regardless of the TMB and MMR/MSI status (147). Compared with the Gem+Cis group, the GC-D group had a median overall survival of 12.8 months versus 11.5 months (hazard ratio [HR], 0.80; 95% confidence interval [CI], 0.66-0.97; p = 0.021), median PFS of 7.2 months versus 5.7 months (HR, 0.75; 95% CI, 0.64-0.89; p = 0.001), and ORR 26.7% versus 18.7% (148). A clinically meaningful and statistically significant survival benefit was reported in treatment-naïve patients with CCA with reference to the combination of two-drug GC-D compared with chemotherapy alone (149). GC-D is expected to be a new first-line treatment option for ICI-based systemic therapy, regardless of the TMB and MMR/MSI status (141).

Gem+Cis is the standard chemotherapy for patients with unresectable iCCA, meanwhile, the combinational strategy of immunotherapy with chemotherapy shows potential against iCCA.

#### 3.2.3 Immunotherapy plus radiotherapy for CCA

Radiotherapy may drive cancer immune alteration *via* several mechanisms, some of which trigger immune suppression, while others trigger immune activation within the TME. A team led by Sharabi and Drake from the Johns Hopkins Department of Oncology found that combining immunotherapy and radiotherapy increases the recruitment of CD8^+^ T cells against tumoral antigens (150). Radiotherapy releases tumor antigens, promotes regulation of immune pathways, increases tumor antigen presentation, initiates tumor-specific cytotoxic T cells, and enhances T cell homing (151). Ionizing radiation may activate the release of the nuclear protein HMGB1 and adenosine triphosphate, described as “damage-associated molecular patterns” (DAMPs), a process that enhances the uptake of tumor-derived antigens by antigen-presenting cells, including neoantigens, caused by RT-driven immunogenic mutations (152).

Stereotactic Body Radiation Therapy (SBRT) is a new radiotherapy method that delivers ablative radiation doses in a limited number of fractions, thereby limiting some of the side effects of traditional radiotherapy. In liver cancers, Kreidieh et al. reported that the combination of immunotherapy and SBRT can improve antitumor immune function by triggering type I IFNs and CD8^+^ T cells and reducing MDSCs in both hepatocellular carcinoma and CCA (153). Although late-stage iCCA with low TMB, microsatellite stable (MSS), proficient mismatch repair (pMMR), and poor PD-L1 expression present ineffective anti-PD-1 monotherapy, the combination of SBRT and ICB retains relative efficacy (154). Liu et al. reported a case of a 68-year-old male with a chemotherapy-resistant stage IV CCA primary tumor with low PD-L1 expression, deficient CD8^+^ cells in the TME, high MSI, and high TMB. The combination of anti-PD-1 immunotherapy and radiotherapy as first-line therapy resulted in the reduction of primary liver tumors and metastatic lymph nodes. Additionally, the combination of radiotherapy and immunotherapy for liver and lung lesions resulted in CR to the primary tumor and all metastases without treatment-related adverse effects (155). Zhao et al. reported four cases of refractory advanced iCCA or pCCA that were successfully controlled with anti-PD-1 antibodies after or in combination with SBRT, meaning that patients achieved CR, PR, or stable disease based on the Response Evaluation Criteria in Solid Tumors (156). In addition, a recent study reported the efficacy of SBRT combined with pembrolizumab in 79 solid tumors, including patients with CCA. Multilocus SBRT plus pembrolizumab was well tolerated and had acceptable toxicity, with a total ORR of 13.2%, a median overall survival of 9.6 months, a median PFS of 3.1 months, and a non-irradiated ORR of 26.9% (157). This indicates a potential for combined therapies to reinforce neoantigen exposure and enhance PD-L1 expression, providing alternative treatments to patients who are non-responsive to radiotherapy or immunotherapy alone (154).

While clinical evidence of the efficacy of immunotherapy and radiotherapy combination treatments in CCA is limited, much of the presented data show great promise (158).

#### 3.2.4 Immunotherapy plus molecular target therapy for CCA

Identification of the drivers of genetic alterations, such as FGFR fusions, isocitrate dehydrogenase (IDH)-1 and -2 mutations, the HER family, neurotropic tyrosine kinase receptor (NTRK) fusions, BRAF mutations, and the Wnt pathway, have contributed to the discovery of more effective targeted therapies (3). Heterozygous mutations in the catalytic arginine residues of IDH-1 and IDH-2 are common in CCA, and the presence of IDH mutations appears to predict better overall survival (159). In patients with advanced IDH-1-mutated CCA that progressed on standard chemotherapy, treatment with ivosidenib, a potent targeted inhibitor of mutated IDH-1, significantly improved PFS and overall survival with a favorable safety profile, after adjusting for crossover (5). A randomized clinical trial (NCT02989857) found that ivosidenib was well tolerated and resulted in a favorable OS benefit vs placebo. The ivosidenib treatment resulted in a median overall survival of 10.3 months in 126 patients who had advanced CCA with IDH-1 mutations and a favorable safety profile (160). According to a systematic review of 5393 CCA cases, approximately 13% of iCCA cases have acquired functional mutations in the IDH-1 coding region (161). IDH catalyzes the conversion of isocitrate to α-ketoglutarate. Alterations in IDH, through accumulation of tumor metabolites, induce extensive epigenetic changes that have pleiotropic effects on differentiation, cell growth, and hypoxia signaling (162). Accumulating evidence suggests that IDH mutations may play an important role in altering the immune TME, manifested by inhibition of TILs, NK cells, and cytotoxic T cells (163). FGFR fusions are observed in approximately 5.7–14% of iCCA cases, and IDH-1 and -2 mutations are detected in approximately 20–30% of iCCA cases. These operable mutations were mainly found in the small ductal-type iCCA. Clinical trials of FGFR2- and IDH-1-targeted therapies have shown promising results (164).

CCAs harboring FGFR2 fusions have recently responded positively to FGFR inhibitors such as pemigatinib, highlighting their potential to be predictive biomarkers (4). However, these agents require further evidence to substantiate their efficacy, and a greater understanding of the ideal genomic subset of patients that would benefit from these treatments is necessary. As a result, other therapies are needed for patients with iCCA who do not respond to FGFR2 inhibitors. Recently, immunotherapy is emerging as a backbone of cancer therapy and is combined with other targeted agents in clinical trials. Chen et al. verified a synergistic effect of anti-PD-1 antibody and lenvatinib in a patient with recurrent iCCA (165). Clinical trials are ongoing to verify the efficacy of the immunotherapy and molecular target therapy combination, with some of them exhibiting promising results in several cancer types (**Table 2**).

With positive results from these trials, the combination of immunotherapy and target-based therapy may have the potential to transform the standard of care for CCA.

#### 3.2.5 Immunotherapy plus antiangiogenesis for CCA

The altered vascularity of the TME limits the circulation of blood and cytotoxic immune cell recruitment, leading to tumor immunosuppression and resistance to immunotherapy. Improving vessel normalization will reverse some of these negative effects, thereby increasing immune cell infiltration and enhancing the efficacy of immunotherapy (166). In hepatocellular carcinoma, Shigeta et al. reported that dual programmed death receptor-1 and VEGF receptor-2 blockade, when administered in combination with various murine models, promote vascular normalization and enhance antitumor immune responses (167). Initial clinical data also support synergistic antitumor activity using the combinational treatment modality. A phase I clinical trial of dual blockade of CTLA-4 (ipilimumab) plus VEGF (bevacizumab) showed increased tumor antigen recognition, tumor-associated endothelial activation, and infiltration of T cells in melanomas (168). Another clinical study revealed that atezolizumab (an anti-PD-L1 antibody) combined with bevacizumab increased the number of intratumoral CD8^+^ T cells and antigen-specific T cell migration (169). Over 100 phase I/II clinical trials are currently testing combinations of anti-angiogenics with immunotherapy (170).

Traditionally, the increase in angiogenesis observed in CCA contributes to its abysmal survival rate after metastasis (171). The therapeutic potential of anti-angiogenesis was recently verified at various stages of development in CCA (3). IMbrave 151 is a randomized, double-blind, placebo-controlled, multicenter, international phase II study on atezolizumab (PD-L1 inhibitor) in combination with chemotherapy (Gem+Cis) and bevacizumab (anti-VEGF monoclonal antibody) as first-line therapy for advanced CCA. It is the first randomized study to evaluate the combination of PD-L1 inhibitor/anti-VEGF antibody to block the chemotherapy center of CCA (172). A combination of anti-angiogenesis and immunotherapeutic interventions that simultaneously perturb both processes may present promising strategies in CCA.

## 4 Discussion

Though considerable novel therapeutic strategies have been assessed, only a limited number of studies have shown promise for CCA therapy due to the intensive recruitment of innate and adaptive immunosuppressive cells, a functional component central to tumorigenesis and tumor progression, particularly in CCA featuring an exuberant desmoplastic reaction. Due to heterogeneity with different gene expression profiles for immune checkpoint pathways (173), the effects of immunotherapy may be limited to small numbers of patients.

Therefore, it is imperative to capture the heterogeneity of the TME among each disease subtype to gain a mechanistic understanding of how each immune cell interacts and functions. It is also critical to define the extent of cellular and molecular heterogeneity of the TME surrounding and infiltrating CCA on treatment outcome and disease progression. High-throughput technologies might provide a considerable amount of information regarding the genetic mechanisms of heterogeneity in CCA (174).

More than 31 studies have been registered on ClinicalTrial.gov to evaluate diverse immunotherapies in CCA patients, yet more preclinical and randomized clinical studies are needed to test immunotherapy in conjunction with other conventional or novel treatments such as chemotherapy, radiotherapy, molecular target therapy, anti-angiogenesis, and anti-lymphangiogenesis to overcome the immunosuppression of CCA. Moreover, with an enhanced understanding of the driver genetic mutations in each CCA subtype, it is critical to identify predictive biomarkers of effective therapy to stratify patients with CCA at the level of disease subtype, genetic drivers, and TME in future trials.

## Author contributions

XZY, LLZ and TW collected the related papers and drafted the manuscript. LLZ, JC revised the manuscript and figures. TW, JC participated in the design, revised, and finalized the manuscript. All authors read and approved the final manuscript.
